# Inter-Annual Variability of Area-Scaled Gaseous Carbon Emissions from Wetland Soils in the Liaohe Delta, China

**DOI:** 10.1371/journal.pone.0160612

**Published:** 2016-08-08

**Authors:** Siyuan Ye, Ken W. Krauss, Hans Brix, Mengjie Wei, Linda Olsson, Xueyang Yu, Xueying Ma, Jin Wang, Hongming Yuan, Guangming Zhao, Xigui Ding, Rebecca F. Moss

**Affiliations:** 1 Key Laboratory of Coastal Wetland Biogeosciences, China Geological Survey, Qingdao Institute of Marine Geology, Qingdao, 266071, China; 2 Laboratory for Marine Geology, Qingdao National Laboratory for Marine Science and Technology, Qingdao, 266061, China; 3 U.S. Geological Survey, Wetland and Aquatic Research Center, Lafayette, Louisiana, 70506, United States of America; 4 Aarhus University, Department of Bioscience and Sino-Danish Centre for Education and Research, Aarhus, C 8000, Denmark; 5 Cherokee Nation Technology Solutions, USGS Wetland and Aquatic Research Center, Lafayette, Louisiana, 70506, United States of America; Saint Francis Xavier University, CANADA

## Abstract

Global management of wetlands to suppress greenhouse gas (GHG) emissions, facilitate carbon (C) sequestration, and reduce atmospheric CO_2_ concentrations while simultaneously promoting agricultural gains is paramount. However, studies that relate variability in CO_2_ and CH_4_ emissions at large spatial scales are limited. We investigated three-year emissions of soil CO_2_ and CH_4_ from the primary wetland types of the Liaohe Delta, China, by focusing on a total wetland area of 3287 km^2^. One percent is *Suaeda salsa*, 24% is *Phragmites australis*, and 75% is rice. While *S*. *salsa* wetlands are under somewhat natural tidal influence, *P*. *australis* and rice are managed hydrologically for paper and food, respectively. Total C emissions from CO_2_ and CH_4_ from these wetland soils were 2.9 Tg C/year, ranging from 2.5 to 3.3 Tg C/year depending on the year assessed. Primary emissions were from CO_2_ (~98%). Photosynthetic uptake of CO_2_ would mitigate most of the soil CO_2_ emissions, but CH_4_ emissions would persist. Overall, CH_4_ fluxes were high when soil temperatures were >18°C and pore water salinity <18 PSU. CH_4_ emissions from rice habitat alone in the Liaohe Delta represent 0.2% of CH_4_ carbon emissions globally from rice. With such a large area and interannual sensitivity in soil GHG fluxes, management practices in the Delta and similar wetlands around the world have the potential not only to influence local C budgeting, but also to influence global biogeochemical cycling.

## Introduction

Wetlands are particularly good locations for sequestering atmospheric carbon (C) through the uptake, transformation, and storage of CO_2_ into plant biomass [1–2]. While the vegetation in many wetlands have high rates of CO_2_ uptake from photosynthesis, anaerobic soils reduce the rate of organic matter decomposition associated with live and dead root fractions, litter and woody debris deposited within the soil, and particulate organic C incorporated into the soil [3–5]. This balance is favorable for storing organic C, and thus potentially providing a natural atmospheric filter for CO_2_-based greenhouse gas emissions (GHG) while simultaneously immobilizing C over long time periods. Indeed, high net primary productivity coupled with reduced decomposition of soil-associated C has jettisoned wetlands to the forefront of scientific curiosity and C legislation as the global atmospheric C pool rises and mitigation is explored [6].

Increases in CO_2_ concentrations in the atmosphere are driven by increased cement production, fossil fuel emissions, and land use change [7], such that concentrations are increasing by 1.9 ppm/year, which equates to perennial increases of 8.6 Pg C/year [8] (1 Pg = 10^3^ Tg = 10^6^ Gg). Given that soil CO_2_ emissions are approximately 68 Pg C/year [9], reducing annual soil CO_2_ emissions from wetlands offer a potential mechanism to help offset atmospheric CO_2_ loading. However, specific CO_2_-reduction management regimes must be identified, targeted, and prescribed on a large scale. Also, management regimes must not facilitate emissions of more deleterious gases, such as CH_4_. In fact, despite a much smaller increase in CH_4_ emissions in recent years [7], CH_4_ still accounts for 25% of the current global warming trend [10]. Wetlands account for approximately 20–25% of the global CH_4_ emissions [11], and scientists and managers are facing difficulties amalgamating the vast number of studies targeting GHG emissions in wetlands [2, 12] to identify better hydrologic, vegetation, and soil management strategies on scales that make a difference.

From a C balance perspective, CO_2_ is the primary molecular source of C into and out of the atmosphere surrounding wetland environments, nearly always being more important than CH_4_ [12–13]. However, because CH_4_ has a greater ability to contribute to global warming than CO_2_, by a factor of 32 [14], a good proportion of wetland studies focus intently on CH_4_ [15–18]. Thus, while wetlands do not emit greater amounts of CH_4_ to the atmosphere than CO_2_ [12], an incremental change in CH_4_ emissions has a disproportionately stronger influence on global warming than a similar shift in CO_2_ [12, 19]. Accordingly, a primary driver of emissions among GHGs over time is land use change and management [20–21].

Shifts in agricultural production of specific crops and irrigation strategies have become a focal point for GHG research in some regions of the world [22–23]. For example, in the Sacramento-San Joaquin Delta of California, USA, agricultural sites managed as drained (pasture, corn field) served as net ecosystem sources of C, emitting 341 g C/m^2^/year of CO_2_ and 11 g C/m^2^/year of CH_4_, while agricultural sites managed as flooded (rice paddy, restored wetlands) served as net ecosystem sinks of C, taking up 397 g C/m^2^/year of CO_2_ while simultaneously releasing a greater proportion of CH_4_ under this hydrologic regime (ranging from 39 to 53 g C/m^2^/year) [24]. CO_2_ and CH_4_ emissions from rice paddy and restored wetlands can vary widely among location based on a number of environmental factors, but often related to water table management [24–27]. Here-in, and for this reason, we focus our current study on gaseous soil C fluxes from different wetland types in the Liaohe Delta of Northeast China to add to a growing body of research that scales assessments spatially [13, 28].

Both the scale of land-use in the Liaohe Delta and year-to-year variation in CO_2_ and CH_4_ fluxes from specific wetlands have the potential to influence regional- and global-scale C cycling [29]. The Liaohe Delta encompasses 5922 km^2^ of natural or managed lands, surrounded or bisected by only 678 km^2^ of towns and roads [30]. Of those natural or managed lands, 55.5% (or 3287 km^2^) are rice paddy (Japonica variety, *Oryza sativa*: 2465 km^2^), reed (*Phragmites australis*: 786 km^2^), seablite (*Suaeda salsa*: 32 km^2^), or mixed communities of reed and seablite (4 km^2^). While rice paddy encompasses the greatest agricultural area in the Delta, over 772 km^2^ of *Phragmites* are managed (compared with 14 km^2^ unmanaged) and harvested annually for paper production [30]. *S*. *salsa* marshes make up the smallest percentage but soils are often associated with high organic C and nitrogen concentrations [31]. Rice paddy and *P*. *australis* wetlands both typically serve as sinks for CO_2_, but emit CH_4_ [24–25, 32], while this course is less clear for *S*. *salsa* marshes [33–36].

Much uncertainty arises when measuring soil CO_2_ and CH_4_ fluxes infrequently or in singular years [26, 34–35], if the goal of such assessment is to upscale to larger areas and over multiple years. Inter-annual and seasonal variations in soil CO_2_ and CH_4_ fluxes are large in coastal wetlands of Northeast China [36–38]. Indeed, we recognized this in a previous study [38], which focused on linking environmental variables (e.g., salinity, soil temperature, water table depth, plant biomass) from a single year to emissions of CO_2_ and CH_4_ from *P*. *australis* marshes, *S*. *salsa* marshes, and rice paddy wetlands of the Liaohe Delta. Here we expand this research to span three very different years from the perspective of hydrology (2012–2014). We had two primary objectives. First, we wanted to know how much C is being lost annually from the soils of these habitat types on an areal basis as assessed using standard discrete sampling techniques. Second, we wanted to assess and discuss drivers of inter-annual variation in emissions of CO_2_ and CH_4_ from three primary wetland types in the Liaohe Delta. While this study does not measure total C balance, we focused intensely on soil C emissions, which are usually the most variable and uncertain component of the C cycle. The linkages among soil C emissions, vegetation type, hydrologic management, season, and spatial coverage of habitats are described across a deltaic region potentially large enough in extent to influence the global C budget.

## Results

### Inter-annual variability of CO_2_ and CH_4_ fluxes

**Fig 1:**
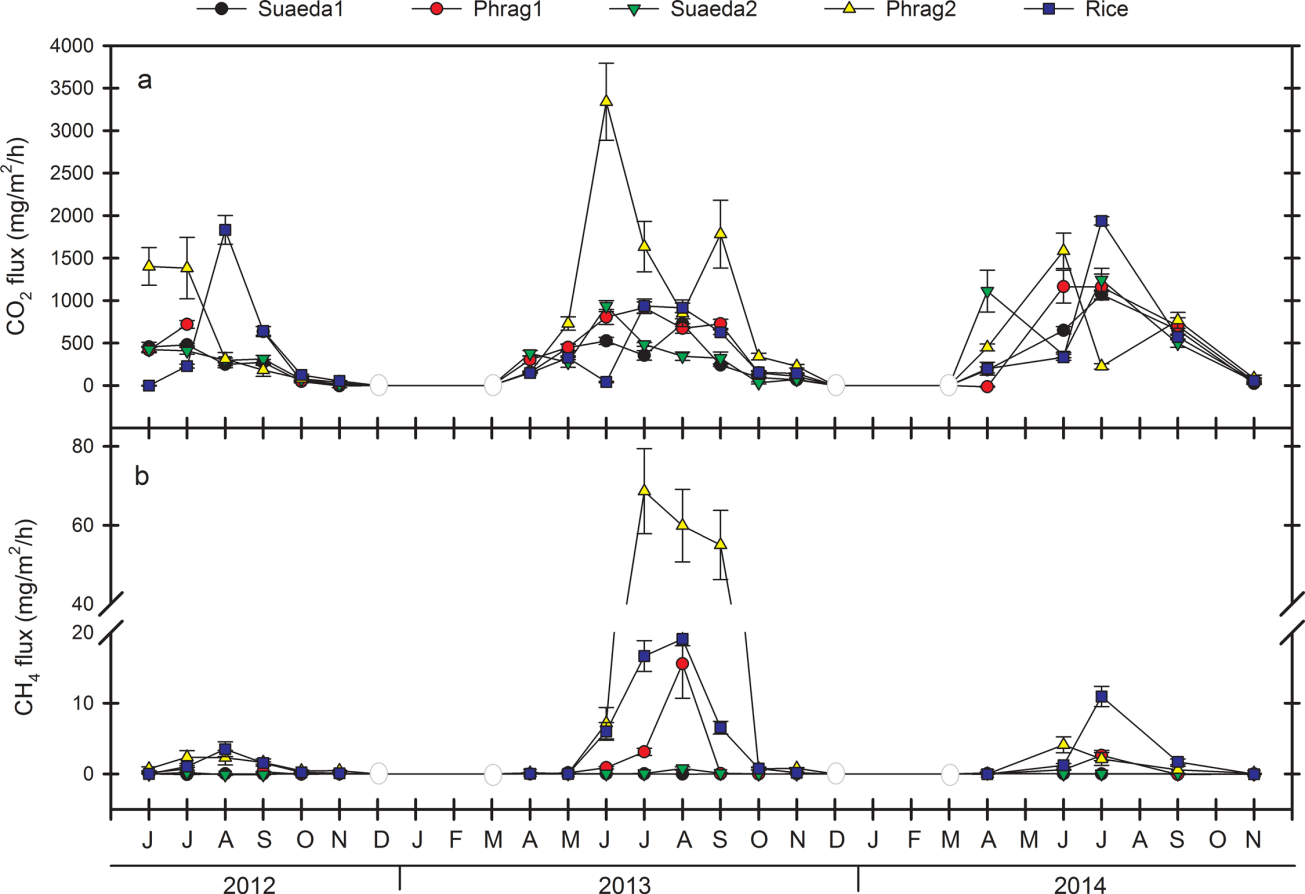
Temporal fluctuation in mean (± 1 SE) soil CO_2_ and CH_4_ fluxes. a) Soil CO_2_ fluxes by sample month over three years, and b) soil CH_4_ fluxes by sample month over three years from five wetland sites (two *Phragmites australis*, two *Suaeda salsa*, one rice) located in the Liaohe Delta, China. Mean values reflect the responses of six replicate chambers per sampling event per site, each canvassing a 55x55 cm soil area. Fluxes were assumed to be zero from December-March when soils were frozen, as depicted by a straight line between open circles.

**Fig 2:**
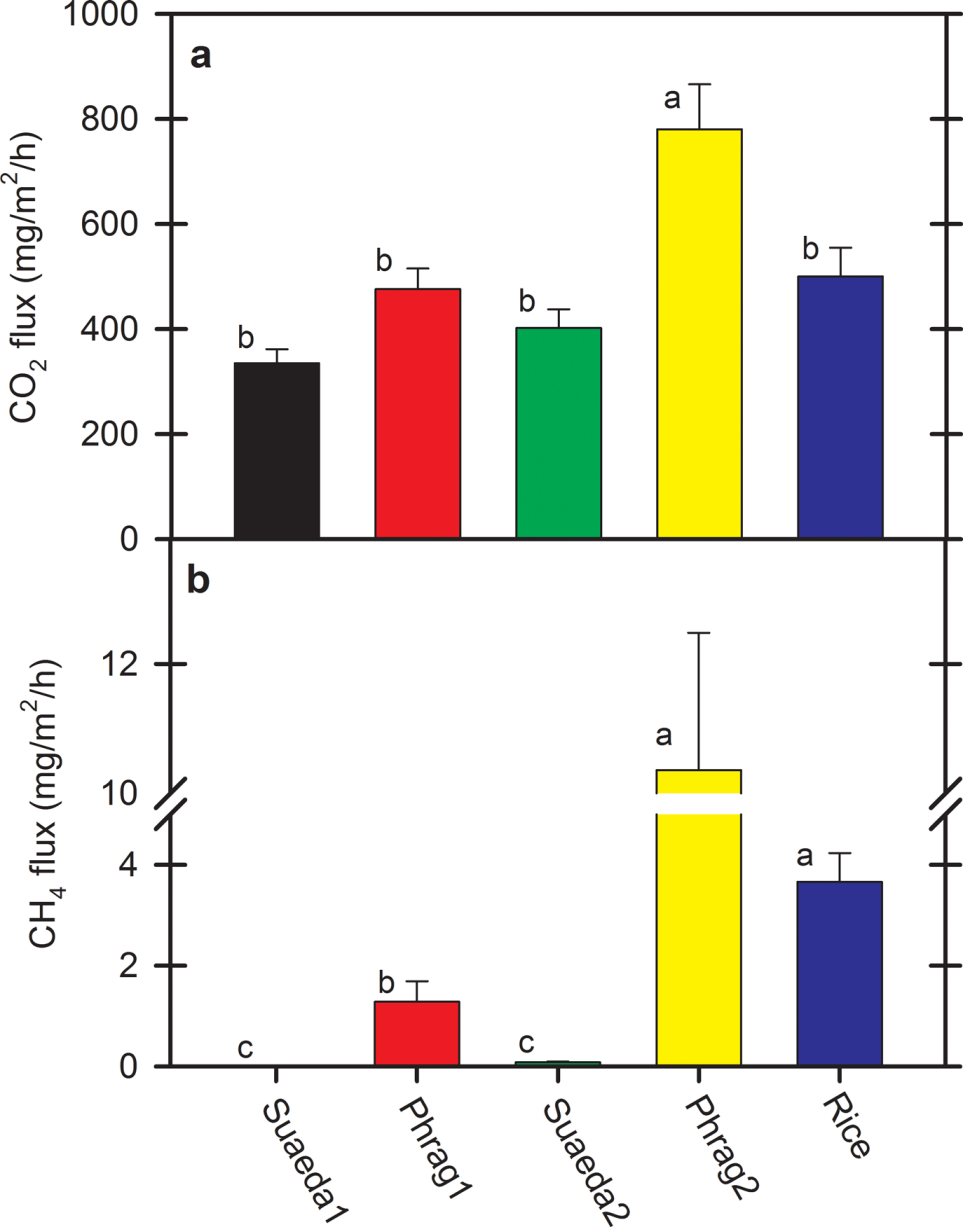
Hourly mean (± 1 SE) soil CO_2_ and CH_4_ fluxes by site. a) Soil CO_2_ fluxes, and b) soil CH_4_ fluxes by site over three years from five wetland sites (two *Phragmites australis*, two *Suaeda salsa*, one rice) located in the Liaohe Delta, China. Means followed by the same letters are not significantly different at α = 0.05. While these site means and differences represent the general trends persisting across all months sampled, a significant site by date interaction (Table 1) limits interpretation when grouped in the fashion.

**Fig 3:**
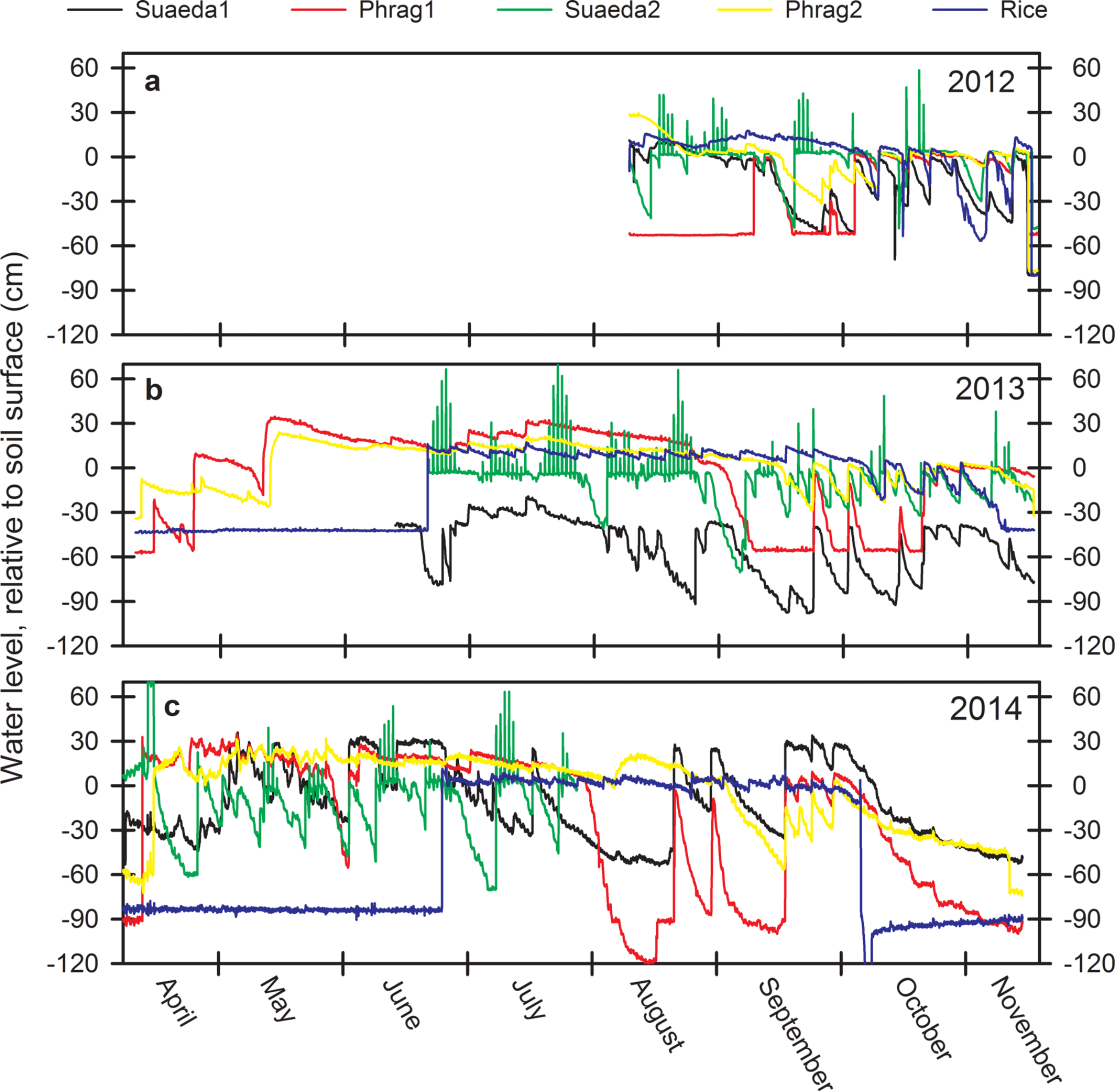
Hydrographs for wetland study sites in the Liaohe Delta. a) Water level patterns for 2012, b) water level patterns for 2013, and c) water level patterns for 2014 from our five wetland sites, including two *Phragmites australis* sites (Phrag1, Phrag2), two *Suaeda salsa* sites (Suaeda1, Suaeda2), and one rice paddy site (Rice) in the Liaohe Delta, China. Missing data from Suaeda1 and Suaeda2 at the beginning of 2013, and for Suaeda2 beginning in August of 2014, represent datalogger failure. Consistent water levels <-30 cm for Phrag1 and Rice indicate times when water levels were below pressure transducers embedded in the soils.

**Fig 4:**
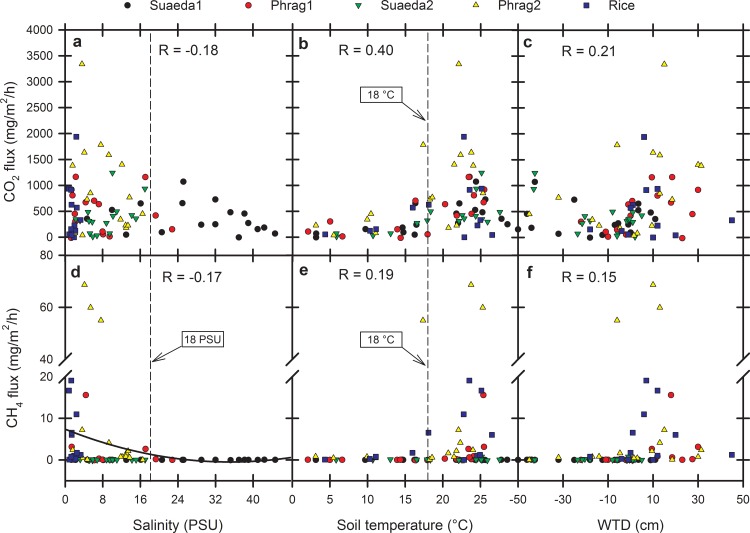
Salinity, soil temperature, and water table depth versus soil CO_2_ and CH_4_ fluxes. a) Soil pore water salinity versus soil CO_2_ flux, b) soil temperature versus soil CO_2_ flux, c) water table depth (WTD) versus soil CO_2_ flux, d) soil pore water salinity versus soil CH_4_ flux, e) soil temperature versus soil CH_4_ flux, and f) water table depth (WTD) versus soil CH_4_ flux by month and site over three years from five wetland sites (two *Phragmites australis*, two *Suaeda salsa*, one rice) located in the Liaohe Delta, China. When present, dashed lines depict important thresholds associated with soil pore water salinity <18 PSU (a, d) and soil temperature >18°C (b, e). For d, the polynomial regression has been re-drawn from [16] and superimposed on data collected from the Liaohe Delta. R = Pearson Correlation Coefficient (P < 0.05 for all).

CO_2_ fluxes varied from year-to-year, especially for Phrag2 and Rice (Fig 1A). Significant site by date interactions highlight this variability (Table 1). For example, while peak soil CO_2_ fluxes were highest in Rice in August of 2012 (1831 mg CO_2_/m^2^/h) and July of 2014 (1937 mg CO_2_/m^2^/h), overall lower fluxes prevailed from that wetland type in 2013 (<937 mg CO_2_/m^2^/h). Capacity for *Phragmites* wetlands to emit CO_2_ from the soil was demonstrated strongly on Phrag2 in 2013 for a single period in June (3339 mg CO_2_/m^2^/h, Fig 1A), and was otherwise fairly consistent among years. CO_2_ fluxes among sites differed from each other on 17 of the 19 dates assessed. Though variable, on average CO_2_ fluxes from Phrag2 (780 mg CO_2_/m^2^/h) were consistently higher (P < 0.05) than Suaeda1 (335 mg CO_2_/m^2^/h), Suaeda2 (402 mg CO_2_/m^2^/h), Phrag1 (476 mg CO_2_/m^2^/h), and Rice (500 mg CO_2_/m^2^/h) (Table 2, Fig 2). A potential driver of inter-annual variability for CO_2_ fluxes from specific sites was hydrology; i.e., Phrag2 and Rice (and the wider region) were flooded for a longer duration in the 2013 growing season from atypical river flooding (Fig 3). Along with water table depth fluctuations, seasonal differences in warming of soils and fluctuations in salinity from year-to-year also influence CO_2_ fluxes on these sites (Fig 4).

**Table 1:** Nested design ANOVA for soil CO_2_ and CH_4_ fluxes over three years from among five wetland sites (two *P*. *australis*; two *S*. *salsa*, and one rice) in the Liaohe Delta, China. DF_num_ = numerator degrees of freedom, DF_den_ = denominator degrees of freedom, MS = Mean Squares, F = F-statistic, P = Probability value (significant if ≤ 0.05).

Gas	Source of variation	DF_num_	DF_den_	MS	F	P
CO_2_	Site	4	25	2.53	8.48	0.0002
	Chamber(Site), *error 1*	25	576	0.30	--	--
	Date	19	455	10.35	96.00	0.0001
	Site x Date	73	455	1.47	13.67	0.0001
	Date x Chamber(Site), *error 2*	455	576	0.11	--	--
CH_4_	Site	4	25	21.73	89.01	0.0001
	Chamber(Site), *error 1*	25	575	0.24	--	--
	Date	19	454	10.03	61.32	0.0001
	Site x Date	73	454	2.72	16.66	0.0001
	Date x Chamber(Site), *error 2*	454	575	0.16	--	--

**Table 2:** Mean CO_2_ fluxes, CH_4_ fluxes, and a suite of physico-chemical characteristics of soils (± SE) from five wetland sites in the Liaohe Delta, China collected over three years.

Variable	Suaeda1	Suaeda2	Phrag1	Phrag2	Rice
CO_2_ flux (mg/m^2^/h)	335 ± 27	402 ± 36	476 ± 39	780 ± 85	500 ± 54
CH_4_ flux (mg/m^2^/h)	0.002 ± 0.008	0.08 ± 0.02	1.28 ± 0.41	10.36 ± 2.12	3.66 ± 0.57
Aboveground biomass (g/m^2^)*	239 ± 18	223 ± 19	308 ± 24	n/a	479 ± 56
Soil temperature (°C)	18.5 ± 0.7	18.5 ± 0.7	17.7 ± 0.7	16.2 ± 0.8	16.7 ± 0.8
Salinity (PSU)	28.5 ± 1.2	8.4 ± 0.4	7.6 ± 0.7	8.9 ± 0.5	1.8 ± 0.1
Water table depth (cm)**	-15.0 ± 2.0	-11.4 ± 1.5	6.3 ± 1.5	3.2 ± 2.1	8.9 ± 1.5
pH	7.87 ± 0.03	8.33 ± 0.01	8.41 ± 0.06	8.23 ± 0.07	8.25 ± 0.03
Porewater HCO_3_^-^ (mg/L)	475 ± 19	393 ± 10	422 ± 23	1369 ± 59	406 ± 32
Soil Eh (mV)	153 ± 74	28 ± 69	145 ± 68	-1 ± 69	103 ± 67

* Commercial harvesting of *P*. *australis* on Phrag2 for pulp prevented consistent estimation of standing biomass.

** Water table depth determined as a mean value from CO_2_ and CH_4_ flux sampling dates.

Significant site by date interactions were noted for CH_4_ fluxes as well (Table 1). In the year receiving more persistent flooding (2013), CH_4_ emissions were 16 times higher from Phrag2 and 6 times higher from Rice than in the other two years (Fig 1B). However, CH_4_ flux increases were not statistically related to water table depth that year (P > 0.5), in contrast to overall correlations among years (Fig 4), suggesting interactive influences with other site variables in 2013. CH_4_ fluxes differed among sites on 15 of the 19 dates assessed. Despite the site x date interaction, CH_4_ emissions from Phrag2 (10.4 mg CH_4_/m^2^/h) were consistently higher (P < 0.05) than emissions from Phrag1, Suaeda1, and Suaeda2 (mean, 0.45 mg CH_4_/m^2^/h, Table 2, Fig 2) on most dates. Variability between the replicate *S*. *salsa* sites was minimal for CH_4_, as salinity kept CH_4_ emissions low at these sites. In contrast, the two replicate *Phragmites* sites behaved very differently; CH_4_ emissions were 8 times higher from Phrag2 than Phrag1 (Table 2).

### Drivers of inter-annual variability

Table 2 depicts means for CO_2_ fluxes and CH_4_ fluxes, along with some environmental variables, as an average of the three years (see S1 Table for additional variables). Of the factors identified as influencing CO_2_ and CH_4_ fluxes in the first year of study [38], most remained unchanged as predictors over three years. Briefly, the overall correlations (across sites) between soil CO_2_ fluxes, soil Eh, soil temperature, aboveground biomass, water table depth, and HCO_3_^-^ concentrations were positive and significant, and soil CO_2_ fluxes were negatively correlated with salinity. CO_2_ fluxes were driven principally by the amount of aboveground plant biomass available to route CO_2_ belowground and through plant tissue, or facilitate microbial soil respiration from exudate production. Thus, to the degree that environmental variables influence plant biomass, they also influence soil CO_2_ fluxes. Direct influences of salinity on CO_2_ emissions were not clear; high variability in CO_2_ emissions at lower salinities (<16 PSU) with reduced variability at high salinity (Fig 4) is confounded by a different vegetation type, *S*. *salsa* versus *P*. *australis*, when salinities exceeded 24 PSU. Otherwise, the capacity for CO_2_ fluxes is reduced in a seemingly linear fashion with salinity. On the other hand and with the exception of one data point from Phrag2, soil temperature limited soil CO_2_ flux to < 690 mg CO_2_/m^2^/h below 18°C. While this is not imposing, much of the cumulative CO_2_ fluxes from all wetland types in the Liaohe Delta occurred as soil temperatures rose above 18°C to a recorded high of 29°C over three years (Fig 4).

The overall correlations (across sites) between CH_4_ fluxes and water table depth, soil temperature, and pore water HCO_3_^-^ concentrations were positive and significant; whereas, the relationship between soil CH_4_ fluxes, Eh, and salinity were negative and significant. There was no significant correlation between CH_4_ emission rates and plant aboveground biomass, which was a little surprising since CH_4_ is often routed through vegetation [15, 17]; but see Materials and Methods about cutting *Phragmites*. Thus for CH_4_, two environmental variables were critical and provided even clearer thresholds than seen for CO_2_ flux when analyzed across sites. Similar to CO_2_, the first limiting variable for CH_4_ flux was soil temperature. The soils of the Liaohe Delta freeze each year, and major fluxes of CH_4_ are not promoted strongly from any wetland type until soil temperatures exceed 18°C (Fig 4). While this was previously suggested [38], the relationship is strengthened by concurrence across the three years of study versus one. The second variable is salinity. This gives way to a second threshold of 18, in that CH_4_ fluxes were widely suppressed (< 1 mg CH_4_/m^2^/h) at soil salinity concentrations >18 PSU across all three years of study (Fig 4). High CH_4_ fluxes were associated with soil temperature >18°C and soil salinity <18 PSU. Thus, wetland management activities in the Liaohe Delta facilitating these two conditions may simultaneously facilitate greater CH_4_ emissions (as long as pore water SO_4_^2-^ availability is also low).

### Emissions of CO_2_, CH_4_, and gaseous carbon from the Liaohe Delta

Approximately 2861 Gg of C is estimated to be emitted from the soils of the Liaohe Delta wetlands annually (Table 3). This value ranges from 2508 to 3285 Gg C/year depending on the year that the estimate was made, and to a lesser degree the specific representative sites used to attain the estimate (Table 3). Site selection was especially important for *P*. *australis* in 2012 and 2013 when Phrag2 had 55% and 129% higher overall C emissions than Phrag1, respectively. When summed, C emissions from CH_4_ were only 1.9% of the C emissions from CO_2_ (Table 3). Emissions of C from CH_4_ equated to approximately 53 Gg C/year, but ranged more broadly from year-to-year for *P*. *australis* wetlands (10–18 times) versus *S*. *salsa* or Rice. Year-to-year variability was less within habitats for CO_2_ emissions, but did vary by up to a factor of 2.6 for specific among year comparisons. Emissions of C from CO_2_ equated to approximately 2808 Gg C/year.

**Table 3:** Accounting of elemental carbon emissions from CO_2_ and CH_4_ fluxes from the soils of three wetland types (five sites) in the Liaohe Delta, China as estimated by year for 2012, 2013, and 2014.

		Gg C/year*						
Variable	Year	Suaeda 1	Suaeda 2	Phrag 1	Phrag 2	Rice	Sum**	Average
CO_2_-C	2012	14.6	14.9	462.4	710.9	1892.2	2493.5	2808
	2013	19.1	20.8	649.2	1423.6	1622.8	2679.1	
	2014	30.2	38.1	770.2	782.2	2441.1	3251.4	
CH_4_-C	2012	-0.0017	0.0055	0.7831	4.5353	11.5582	14.2	53
	2013	-0.0002	0.0239	8.5291	82.8640	66.5809	112.3	
	2014	-0.0008	0.0066	2.1701	4.7632	29.8900	33.4	
**Total C**	2012	14.6	14.9	463.1	715.4	1903.7	2507.7	**2861**
	2013	19.1	20.8	657.8	1506.4	1689.4	2791.4	
	2014	30.2	38.1	772.4	787.0	2471.0	3284.8	

* Based on a total land area of 31.6 km^2^ for *Suaeda salsa*, 786 km^2^ for *Phragmites australis*, and 2464.6 km^2^ for Rice [30]; Gg = 10^9^ grams

** Weighted sum across habitats; average of both *S*. *salsa* sites, plus average of both *P*. *australis* sites, plus Rice.

## Discussion

### Temporal scale and variability

Variability in gaseous C emissions from wetlands as a component of the mass C balance is important to consider when determining whether specific wetlands experience net gains, emissions, or steady state fluxes of C over time. For this reason, many studies link environmental drivers (e.g., soil temperature, water level, salinity, etc.) to CO_2_ or CH_4_ emissions with the intent of using statistical relationships to predict losses or gains [15, 18, 33, 39–41]. Yet, statistical relationships developed in a single year can fail to predict CO_2_ or CH_4_ emissions accurately in years having different magnitudes of response. For example, actual emissions of CH_4_ were much greater from *P*. *australis* wetlands in the Liaohe Delta in 2013 than either 2012 or 2014. While water table depth, soil temperature, and salinity were important in all years as similarly established for 2012 [38], regressions developed for 2012 would have underpredicted CH_4_ emissions in 2013. Assessment variability is a dilemma in the prediction of GHG emissions at large scales. For example, North American wetlands emit approximately 9.4 Tg of CH_4_/year (or about 7 Tg C/year), but uncertainty around this value is up to 100% [2]. Indeed, C emissions from CH_4_ were over an order of magnitude higher from Phrag1 and Phrag2 in 2013 versus 2012 and 2014, and C emissions from CO_2_ from Suaeda1 and Suaeda2 in 2014 were nearly double emissions in 2012 and 2013 (Table 3).

To compound this further, GHG techniques measure vastly different things [42]; e.g., compare large dark static flux chambers (30,250 cm^2^) incorporating vegetation (as used here) versus small static chambers (80 cm^2^) that exclude vegetation versus eddy covariance, which measures the net ecosystem exchange of C over many hectares. While we standardize our sampling area and techniques among years, year-to-year CO_2_ fluxes varied by a factor of up to 2.6 and CH_4_ fluxes varied by a factor of up to 18 in the Liaohe Delta within a specific wetland type. Similar trends were reported from the nearby Yellow River Delta, where complete reversals of CO_2_ and CH_4_ fluxes from soil uptake to efflux were documented for some wetland types (e.g., *P*. *australis*) among years [36], although the reasons were not discussed. Indeed, we can conclude that GHG assessments across multiple years are critical for determining mass C fluxes from wetlands.

### Factors influencing area-scaled CO_2_ and CH_4_ emissions

On average, 28.0% of the C emissions from CO_2_ were derived from *P*. *australis* wetlands (800 Gg C/year) and 71.2% were derived from rice (1985 Gg C/year), leaving only 0.8% associated with *S*. *salsa* wetlands (23 Gg C/year) (Table 3). These differences are compounded mostly by the areal extent of each wetland type. Statistically, CO_2_ flux from only one *P*. *australis* wetland (Phrag2) was consistently greater than the two *S*. *salsa* sites and rice when standardized over a square meter area (Table 2; Fig 2). Noteworthy among the different sites was the much greater C and nitrogen density in the soils of Phrag2 that may be influencing high CO_2_ fluxes (S1 Table). Therefore, while aboveground biomass was undetermined for Phrag2 versus other sites (Table 2), the availability of C-based substrate within the soil to facilitate microbial respiration was over two times greater on Phrag2 than even Phrag1. The high proportion of soil organic C on this one *P*. *australis* site may be related to either the vegetation type itself or the particular water/harvest management influencing that site.

The relative proportion of labile soil C in *S*. *salsa* soils of the Liaohe Delta was influenced greatly by the presence of vegetation [43]; in that case, bare soils versus *S*. *salsa*-vegetated tidal flats. *P*. *australis* plants are much larger than *S*. *salsa*, and have a strong ability to sequester C by maintaining high aboveground and belowground plant biomass [32]. Persistent flooding would also keep soils anaerobic and further limit decomposition; both *P*. *australis* sites were often flooded and maintained water tables above ground during sampling (Table 2). Annual commercial harvesting of *P*. *australis* for pulp production in the Liaohe Delta compromises the role that perennial pulses of litterfall would play in facilitating nutrient recycling in this wetland type, and perhaps even upset some biogeochemical processes spatially across the Delta adding further to variation in CO_2_ (and CH_4_) emissions from *P*. *australis*.

On average, 32.4% of the C emissions from CH_4_ were derived from *P*. *australis* wetlands (17.3 Gg C/year) and 67.5% were from rice (36.0 Gg C/year). What was slightly different for CH_4_ versus CO_2_, was that only ~0.01% of the C emissions from CH_4_ was associated with *S*. *salsa* wetlands (0.006 Gg C/year) (Table 3). For *S*. *salsa*, suppression of CH_4_ was due to a combination of smaller areal extent and potentially greater SO_4_^2-^ availability in the porewater [44]; salinity concentrations were above 45 PSU at times (average of 28.5 PSU for Suaeda1, Table 2) and water tables were either maintained below ground through impoundment (Suaeda1, with the exception of 2014) or were tidal (Suaeda2) (Fig 3).

As previously suggested in a global review [16], salinities above 18 PSU also tended to limit CH_4_ emissions from the Liaohe Delta; a regression superimposed on the salinity versus CH_4_ flux relationship from the Liaohe Delta indicates the fit suggested previously [16], and is remarkably applicable here when applied to three years of data collection across all wetland types in the Liaohe Delta (Fig 4D). Also important is that a reduction of salinity from 7.9 to 3.1 PSU over the growing season (May-September) from the two *P*. *australis* sites and rice site in combination gave rise to a 13-fold increase in average CH_4_ fluxes in 2013 versus 2012 (17.3 vs. 1.3 mg CH_4_/m^2^/h, respectively). SO_4_^2-^ delivery to soils at low salinity is associated with high spatial variability in SO_4_^2-^ suppression [16, 38]; this is a three-dimensional variability in space, making larger chambers necessary for capturing net flux changes, such as these, over larger areas when salinity concentrations are low.

The course of CH_4_ suppression is less clear for the second *S*. *salsa* site (Suaeda2), which had a mean salinity of only 8.4 PSU. Oddly, this salinity concentration was well within the salinity ranges of both *P*. *australis* sites (7.6–8.9 PSU), yet both *P*. *australis* sites maintained high CH_4_ emissions. Higher salinity in the upper soil layers would certainly influence CH_4_ emissions on *P*. *australis* sites less because much methanogenesis occurs deeper where anaerobic soil layers persist and soil pore water would be fresher. CH_4_ might then route from deeper-laying *P*. *australis* roots, through stem tissue, and released to the environment providing a CH_4_ conduit from lower, oxygen-deficient freshwater layers that bypass salinity influence and soil layers with an active methanotrophic bacterial community [11, 45]. Deep roots and rhizomes may make all the difference for *P*. *australis*, relative to *S*. *salsa* wetlands. Over the first year of study [38], low CH_4_ emissions from Suaeda2 were linked to the same SO_4_^2-^ suppression mechanism observed for Suaeda1, since salinity ranged to 15 PSU at times and salinity was probably pulsed higher at other times missed by our sampling. Suaeda2 is also strongly tidal compared to all of the other sites (Fig 3), and exposed soils during low or neap tides would facilitate CH_4_ oxidation to CO_2_. Fluctuating water tables may also help explain lower CH_4_ at this *S*. *salsa* site (Suaeda2) as the capacity for CH_4_ oxidation is greater as soils are more exposed [46]. Multi-year, area-scaled assessments that isolate *P*. *australis* or *S*. *salsa* to assess influence from additional wetlands in China are not available. However, one smaller effort from the Yellow River Delta provides some guidance [35]. There, the smaller area of *P*. *australis* wetlands assessed (88.1 km^2^ in the Yellow River Delta vs. 786.0 km^2^) and a larger area of *S*. *salsa* wetlands assessed (90.2 km^2^ in the Yellow River Delta vs. 31.6 km^2^) suggested that C emissions from the Yellow River Delta were much less (59.2 Gg C/year) than we reported from the Liaohe Delta (2861 Gg C/year).

CO_2_ fluxes from rice paddy soils were large across the Delta (Table 3), although CO_2_ emissions from soils may be balanced by, or less than, uptake of CO_2_ by photosynthesis in such a productive environment. Indeed, the same notion (i.e., > CO_2_ gains vs. emissions) may hold for all three wetland types. For example, based on *P*. *australis* photosynthesis data previously reported [32], *P*. *australis* wetlands in the Liaohe Delta would fix approximately 1,600 Gg C from atmospheric CO_2_ annually [30]. Based on our data, C from soil CO_2_ emissions would range from 39–61% of that value across the Delta, suggesting large-scale C sequestration among *P*. *australis* wetlands in the Liaohe Delta despite large soil emissions of CO_2_. Adding CH_4_ affects the balance for C by only a small amount for *P*. *australis* (add 0.24–1.9% to the percentages for CO_2_). More quantitatively, rice paddies in California, USA had a net ecosystem uptake of 50–397 g C/m^2^/year from CO_2_ [24–25]; CH_4_ emissions from these same sites in California were also quite low (2.5–6.6 g C/m^2^/year [25]) to moderate (39–53 g C/m^2^/year [24]). For comparison, Liaohe Delta rice paddies registered CH_4_ emissions of 4.7–27.0 g C/m^2^/year when scaled annually.

Higher CH_4_ fluxes from rice may be explained, in part, by hydrologic management. Our measured fluxes of ~4 mg/m^2^/h (Table 2) were much smaller than other rice fields under continuous irrigation in China (mean ± SD, 13.6 ± 9.2 mg/m^2^/h [47]). These literature values for CH_4_ emissions are 64% higher than rice cultivated under drier, intermittent irrigation (mean ± SD, 8.3 ± 7.7 mg/m^2^/h [47]). Water levels were maintained well above the soil surface for most of the active cultivating season (June to September) for all three years in the Liaohe Delta, averaging 13.4 (± 3.7 SE) cm above ground. Not all Chinese rice paddies are managed in this fashion [47]; studies have indicated that mid-season drainage of rice paddies can reduce CH_4_ emissions by 36–65% [48]. Such hydrologic management is at least feasible across many hectares of the Liaohe Delta owing to the “square-land method” [30], such that individual landowners could theoretically regulate CH_4_ emissions at a local scale. However, prescribing drained or moist-soil management versus persistent flooding regimes is not simple to implement based solely on univariate relationships. Rice paddies are often loaded with NO_x_-based fertilizers such that drainage may mitigate emissions of C from CH_4_ (i.e., a rather small component of the C flux, as we show here), but greater exposure to oxygen during drainage might simultaneously facilitate denitrification of NO_x_ and promote N_2_O emissions when denitrification is incomplete. NO_x_ is often combined with surplus soil acetate from crop residue by chance of timing during drainage. N_2_O has an even higher radiative forcing value than CH_4_; six times higher than CH_4_ when modelled as sustained-flux global warming potentials over a 100-year time frame [19].

### Global perspective of C-based soil GHG flux

Soil CO_2_ emissions from all ecosystems globally is approximately 68 Pg C/year (± 4 SD) [9] (1 Pg = 10^3^ Tg = 10^6^ Gg), and while uncertain, soil CO_2_ fluxes can also be high for wetlands [13]. As we describe here-in, soil emissions are often balanced by, or are lower than, net ecosystem uptake of CO_2_ in order for atmospheric C to be sequestered by wetlands. Unless wetlands are deteriorating or are unhealthy, C sequestration is a strong characteristic of wetlands, which are estimated to serve as C sinks for 0.83 Pg C/year globally [12]. Soil C emissions of CO_2_ from wetlands across the Liaohe Delta were estimated as 2.8 Tg C/year (Table 3). More important, this value tended to fluctuate among years from 2.5–3.3 Tg C/year, suggesting a strong potential year-to-year influence from wetland management or from stochastic environmental fluctuations.

Adding CH_4_ to this estimate makes very little difference from a C emissions perspective, affecting emissions by ~0.05 Tg/year at that resolution (Table 3). However, this is not to say that CH_4_ is unimportant. In fact, as we describe, wetland management that facilitates lower salinity (below 18 PSU) and a quicker seasonal return to soil temperatures of 18°C or greater (as practiced in the Liaohe Delta [30]), would influence CH_4_ fluxes considerably. This was the case in 2013, when CH_4_ fluxes were higher from *P*. *australis* and rice due to persistent flooding [49] and salinity reduction. A focus on radiative forcing from CH_4_ [12–13, 19] versus total C emissions may provide a different perspective from the Liaohe Delta. Furthermore, other studies have discovered CH_4_ emissions from rice growing in Northeast China to be even higher than we reported here [47], and our sampling would have missed any pulsed CH_4_ emissions due to annual thawing [50].

Estimates of global C emissions from CH_4_ for natural wetlands range from 69 to 213 Tg C/year [19, 51–52] and for rice paddies range from 25 to 30 Tg C/year [19, 52], with an average of 162 Tg C/year and 27 Tg C/year for natural wetlands and rice paddies, respectively [19]. Since CH_4_ is not normally taken up by wetland soils through biological activity, flux values reported here-in would approximate the true gaseous C balance for CH_4_. Caveats do apply, such as small fluxes of CH_4_ into the soil due to pressure differentials [53]. For rice, we also discovered that lower pore water HCO_3_^-^ concentrations corresponded to higher CH_4_ fluxes (P < 0.001, r = -0.49), suggesting that whatever is facilitating higher CH_4_ fluxes (e.g., anaerobiosis) may be reducing pore water HCO_3_^-^ by influencing dissolution of CO_2_. Overall, our total estimate of 0.053 Tg C lost per year to CH_4_ emissions from all Liaohe Delta wetlands assessed is also seemingly low, except that emissions from Liaohe Delta rice paddies alone make up approximately 0.2% of rice paddy CH_4_-C emissions globally. For scale, C emissions from CH_4_ for *Carex lasiocarpa*-dominated peatlands spread out over a much larger area in China’s Sanjiang Plain to the north of the Liaohe Delta was estimated to be lower, at 0.007 Tg C/year [54], than we report from rice.

## Conclusions

*Phragmites australis*, *Suaeda salsa*, and rice paddy wetlands encompass an area of approximately 3,287 km^2^ in the Liaohe Delta, China. This is the world’s largest continuous *P*. *australis* wetland and China’s third largest oil field, and the Delta produces a large percentage of the rice crop for China in a given year. Total C emissions from CO_2_ and CH_4_ from these wetland soils average 2.9 Tg C/year, but range from 2.5 to 3.3 Tg C/year. We surmise that hydrology by way of management (e.g., longer retention times for water held within impoundments) or natural variability (e.g., rainfall and regional flood patterns) was a primary inter-annual driver of these differences, suggesting that evaluations of greenhouse gas fluxes need to be framed over multiple years. The primary emissions of gaseous soil C were from CO_2_ (~98%). While photosynthetic uptake of CO_2_ would most often overwhelm CO_2_ emissions from the wetland soils as they build aboveground and belowground C stores, CH_4_ emissions would persist. Overall, the opportunity for higher CH_4_ fluxes was associated with soil temperatures >18°C and pore water salinity <18 PSU. CH_4_ emissions from rice paddy habitat alone in the Liaohe Delta represent 0.2% of total C emissions from CH_4_ globally for that habitat type. With such a large area and apparently sensitive feedbacks with soil CO_2_/CH_4_ fluxes on a year-to-year basis, management practices in the wetland area studied and similar wetlands around the world have the potential not only to influence local C budgeting, but also to influence global biogeochemical cycling.

## Materials and Methods

### Ethics statement

The Panjin Wetland Science Research Institute (Mr. Dechao Sun, director) granted permission to access sites Phrag1, Phrag2, Suaeda1, and Suaeda2, and Mr. Tiejin Li granted permission to access the Rice site within his village.

### Study sites

The Liaohe Delta is located in Liaoning Province in Northeast China, and has a geomorphic connection to four rivers; the largest is the Liaohe River. The Liaohe River is 1396 km long with a drainage area of 219,000 km^2^, and contemporary agricultural and deltaic wetland area of 3606 km^2^, encompassing the world’s largest reed field, expansive rice paddies, and intertidal and unvegetated wetlands [30]. Polluted river waters [55] and active oil and gas mining activity (as China’s third largest oil field [56]) pose significant environmental hazards for the Delta; river water is incredibly important for wetland irrigation while industrial canals, pipelines, and oil and gas mining infrastructure have transformed the landscape. Management of wetlands involves the use of pumping stations to divert Liaohe River water to *P*. *australis* wetlands to desalinize stands, thaw soils earlier in the growing season, and buffer soils from re-freezing nightly to promote greater productivity. Indeed, this action helped to increase *P*. *australis* yield to the pulp industry by ~137,000 metric tons over a 31 year period up to 1980 [30]. Local-scale hydrologic management (“square-land method”) was implemented intensely both for rice and *P*. *australis*, while *S*. *salsa* marshes typically exist as natural tidal features farther down the Liaohe River but are sometime impounded.

**Fig 5:**
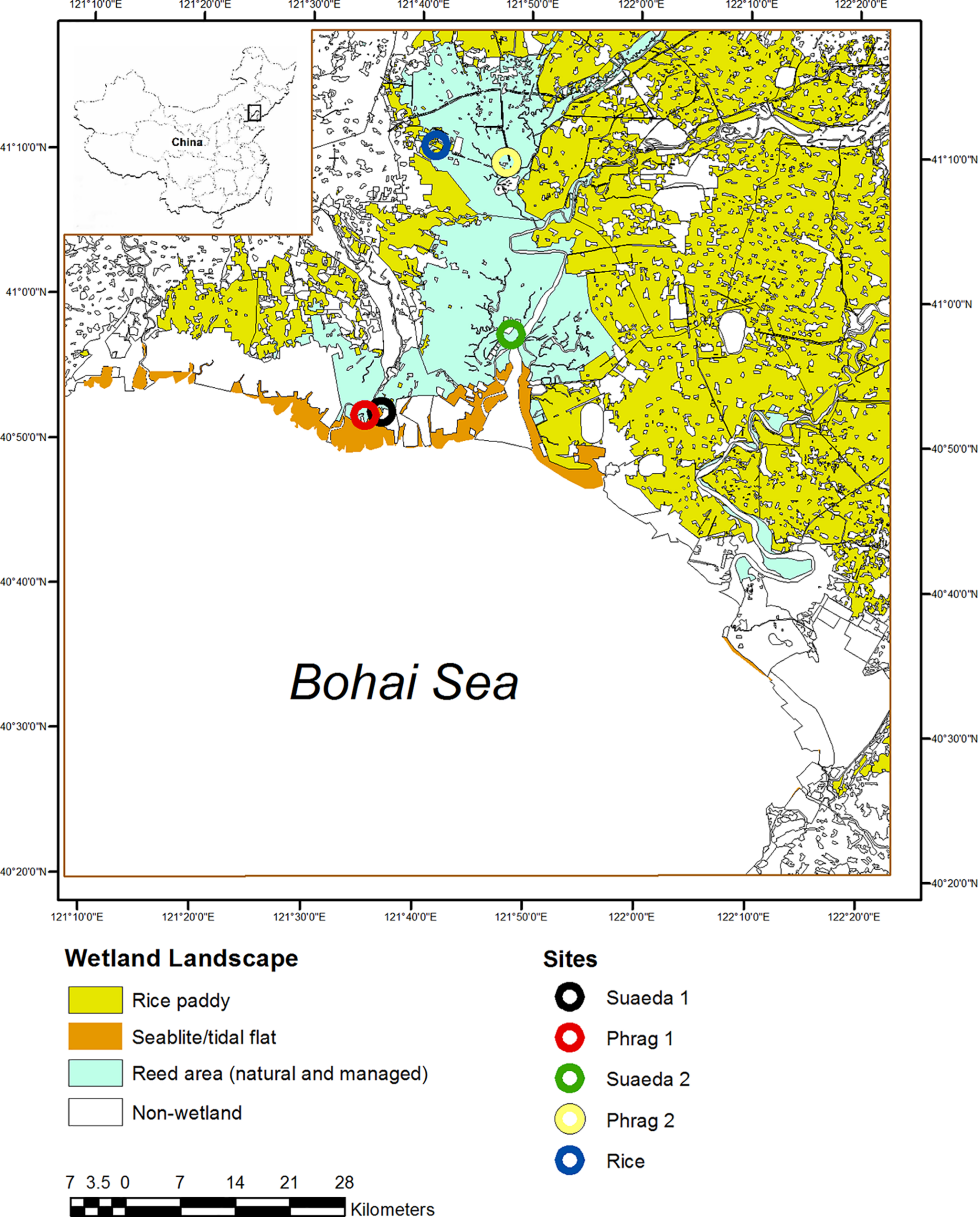
Location of study sites and aerial distribution of habitat types sampled in the Liaohe Delta, China. Map highlights 31.6 km^2^ of *Suaeda salsa* wetlands, 786 km^2^ of *Phragmites australis* wetlands, and 2464.6 km^2^ of rice paddy wetlands, as well as the location of our five wetland sites, including two in *Phragmites australis* (Phrag1, Phrag2), two in *Suaeda salsa* (Suaeda1, Suaeda2), and one in rice paddy (Rice). Aerial distribution data are from [30], and the shape file represents 2011 classifications (China Geological Survey).

Five sites representing the three primary wetland types in the Delta were selected (Fig 5). Two sites included managed reed (*Phragmites australis* (Cav.) Trin. Ex Steud.) wetlands (“Phrag1” at 40° 52’22.34”N, 121°36’08.89”E; “Phrag2” at 41° 09’33.75”N, 121°47’42.71”E) for paper production, two sites included seablite (*Suaeda salsa* (L.) Pallas) wetlands (a created and semi-impounded “Suaeda1”, 40° 52’11.09”N, 121°36’21.72”E; a natural “Suaeda2”, 40° 57’38.62”N, 121°48’20.03”E), and one site had active rice (*Oryza sativa* L.) agriculture (“Rice”, 41° 10’38.69”N, 121°41’17.28”E). Sites were selected carefully and over many days of searching to be representative of those wetland types in the wider region. With the exception of Phrag2, soil properties were fairly consistent among sites (S1 Table).

The air temperature in the region associated with the Liaohe Delta ranges from an average low of –10.4°C in January to an average high of 27.4°C in July, with an annual average of 8°C and approximately 175 days/year frost-free [43]. The annual precipitation for the Delta is 612 mm [43]. Remarkably, the year 2013 tied with 2007 as the sixth warmest since global records began in 1850 [49]. 2013 was also warmer than both 2011 and 2012, which, though marked by cooling La Niña conditions, were 0.43°C and 0.46°C above average, respectively [49]. In addition to high temperatures in 2013, anomalous hydro-meteorological events affected northeastern China with excessive river flooding [49] with noticeable impacts to the Liaohe Delta seasonally relative to 2014 in terms of more persistent flooding on study sites, especially for Phrag1, Phrag2, and Rice.

### Experimental design and GHG flux measurements

Soil CO_2_ and CH_4_ gas fluxes were sampled approximately monthly from June to November for Year 1 (2012), April to November for Year 2 (2013), and April to November for Year 3 (2014). Gases were collected using six, square metal frames installed permanently on 4 of 5 sites. Frames had to be moved annually to accommodate agricultural activity on one site (“Rice”). Frames had an area of 3025 cm^2^ (55x55 cm), were constructed with small drain holes at the base to allow free water flow between measurement periods, and had troughs for inserting white, plastic chamber tops during sampling. Holes were plugged, troughs were filled with water, and the chamber tops were lined internally with aluminum foil to ensure that light would not penetrate into the “dark” chambers during sampling. Chamber tops were 30 cm tall, requiring that *P*. *australis* plants were cut at times; however, we limited cutting to only as much as necessary to emplace chamber tops. This practice had very little influence on CH_4_ emissions when reeds were cut above standing water [45], as we practiced here. All chambers were accessed from permanent boardwalks positioned just about the soil surface.

For Year 1, gases were sampled using static flux chamber protocols [57]. Tops were emplaced and gases were extracted through rubber septa using a 15 mL syringe, and injected into pre-vacuumed 10 mL glass vials for analysis on a laboratory based gas chromatograph (GC). Samples were taken as soon as the chamber tops were emplaced, and at 20 min intervals over 60 mins. Circulating fans kept gases mixed within chambers, which were approximately 121 L in size with chamber tops emplaced. Full sampling details for Year 1 including GC information, storage and laboratory protocols were previously provided [38].

For Years 2 and 3, a portable GC (Model 915, Los Gatos Research, Mountain View, CA, USA) was used instead of a laboratory based GC in order to facilitate in-situ measurements and overcome any concerns we had in Year 1 with storing and transporting gas vials over 520 km from the Liaohe Delta to Qingdao. The chamber tops were the same as for Year 1, but septa were replaced with Tygon tubing routed to and from the portable GC. For both methods, flux rates were determined using the linear portion of fit saturation curves comparing static flux over time (Year 1) or steady state flux rate increases over time (Years 2 and 3). All samples were taken during the day and assumed to be consistent diurnally for that day, but see [58–59].

### Soil characteristics

Soil cores (3 per site) were taken to a depth of 10 cm, extracted by pushing/twisting a 15-cm diameter by 1-m long metal cylinder (0.8-mm-wall) with a sharpened end into the soil with minimal compaction, and sectioned into 2 cm increments. 2-cm sections were mixed thoroughly, dried to a constant weight at 60°C, and ground. Soil bulk density, water content, and pH were determined through standard procedures, and nitrogen and carbon (total and organic) were then analyzed. Individual samples were split, with total nitrogen and total C analyzed on one section with a CHNS/O elemental analyzer (2400 Series, Perkin Elmer, Waltham, MA, USA). The second section was used to determine organic C fractions on the same elemental analyzer, but after inorganic C was removed with 4 M HCl [60]. Sections (*n* = 5) were averaged after analysis for each core.

Soil oxidation-reduction potentials (Eh) were determined with brightened platinum electrodes inserted to a depth of 10 cm [61], and allowed to sit for 24 h prior to measurement to ensure a well-poised couple. Eh probes were referenced against calomel electrodes, and adjusted by adding 245 mV for standardization against a hydrogen electrode scale. Water level recorders (model 3001, Solinst, Georgetown, Ontario, Canada) were inserted into on-site wells during freeze-free periods, and recorded water table depth hourly. During sampling, salinity was measured from temperature-compensated conductivity on water extracted from on-site piezometers using a meter (Model 6010, Jenco Electronics, Ltd., Shanghai, China), and soil temperature was measured using manual thermometers (bi-metallic dial, H-B Instruments, Collegeville, PA, USA) inserted to a 10-cm soil depth just outside of each static flux chamber. Plant aboveground biomass was sampled monthly to coincide with gas flux measurements seven times each in 2012 and 2013 (May to November) and 4 times in 2014 (April, June, July, September) from Phrag1, Suaeda1, Suaeda2, and Rice using 55 cm × 55 cm frames (*n* = 6/site). Phrag2 was sampled identically when feasible; however, commercial harvesting of *P*. *australis* for pulp from that site prevented consistent biomass estimates. All vegetation within the frame was clipped at the soil surface, dried to a constant weight at 60°C, and weighed.

### Statistical analysis and variability determinations

Soil CO_2_ and CH_4_ emissions were analyzed with ANOVA in a split-plot framework using Type IV sums of square error estimation for accounting for missing treatment combinations. Date was assigned as a whole-plot effect (repeated measures). There were a total of 19 monthly CO_2_ and CH_4_ flux assessments over the 3 years, but only five sites. For repeated measures analyses, the assumption of n+1>q (where n equals the sample size, i.e., number of sites, and q the number of repeated measures) was not met [62], so we nested terms to account for non-independence among repeated measures [41, 63]. For significant treatment by date interactions, treatment differences were determined with Bonferroni adjustment. All data were log-transformed. The errors had a homogeneous variance and were unimodal and symmetric. Correlation analysis was used to determine whether gas fluxes and soil water table, salinity, above ground biomass, porewater HCO_3_^-^, Eh, or soil temperature related over a three year period. Data were analyzed using SAS (Version 9.3, SAS Institute, Cary, NC, USA).

Average annual rates and variation of CO_2_ and CH_4_ emissions were determined from each site for each year, and scaled assuming: (1) that mean hourly rates of CO_2_ and CH_4_ emissions from chambers are consistent over a measurement day, (2) that days sampled over the course of individual years (n = 5–8 times/year) are representative of the year, and (3) that no fluxes occurred when soils were frozen (December, January, February, March). Soils in the Liaohe Delta freeze solid to depths of > 0.5 m in the winter. Based on near-zero fluxes in November of every year (Fig 1), this latter assumption appears valid (but see [50, 64] for CH_4_ emissions). We recognize that measurements are not continuous over individual years, but we wanted to document how commonly used discrete sampling procedures can reveal inter-annual differences in important GHG fluxes related to a combination of site management and environmental factors. Mean fluxes from Phrag1/Phrag2, Suaeda1/Suaeda2, and Rice were reduced to three values each for CO_2_ and CH_4_, and multiplied over area determinations from 2009 satellite imagery for the Liaohe Delta [30].

## Supporting Information

S1 Table Soil characteristics from a depth of 0–10 cm at five wetland sites in the Liaohe Delta, China. Data are updated from [34] to include additional data collected in 2013 and 2014.
(DOCX)

S2 Table Raw data used for interpretative purposes in “Inter-Annual Variability of Area-Scaled Gaseous Carbon Emissions from Wetland Soils in the Liaohe Delta, China”. (XLSX)

## References

[1] Mcleod E, Chmura GL, Bouillon S, Salm R, Björk M, Duarte CM, et al (2011) A blueprint for blue carbon: toward an improved understanding of the role of vegetation coastal habitats in sequestering CO_2_. Front. Ecol. Environ. 9: 552–560.

[2] Bridgham SD, Megonigal JP, Keller JK, Bliss NB, Trettin C (2006) The carbon balance of North American wetlands. Wetlands 26: 889–916.

[3] Chmura GL, Anisfeld SC, Cahoon DR, Lynch JC (2003) Global carbon sequestration in tidal, saline wetland soils. Glob. Biogeochem. Cycles 17: 1111.

[4] Middleton BA, McKee KL (2001) Degradation of mangrove tissues and implications for peat formation in Belizean island forests. J. Ecol. 89: 818–828.

[5] Donato DC, Kauffman JB, Murdiyarso D, Kurnianto S, Stidham, et al (2011) Mangroves among the most carbon-rich forests in the tropics. Nat. Geosci. 4: 293–297.

[6] Duarte CM, Losada IJ, Hendriks IE, Mazarrasa I, Marbà N (2013) The role of coastal plant communities for climate change mitigation and adaptation. Nat. Clim. Change 3: 961–968.

[7] Ciais P, Sabine C, Bala G, Bopp L, Brovkin V, Canadell J, et al (2013) Carbon and other biogeochemical cycles, in *Climate Change 2013*: *The Physical Science Basis*, edited by Stocker T.F., Qin D., Plattner G. -K., Tignor M., Allen S. K., Boschung J., Nauels A., Xia Y., Bex V., and Midgley P. M., pp. 465–570, Cambridge, UK and New York, NY, USA: Cambridge University Press.

[8] Lal R (2008) Carbon sequestration. Phil. Trans. Royal Soc. B 363: 815–830. 10.1098/rstb.2007.2185 PMC2610111 17761468

[9] Raich JW, Schlesinger WH (1992) The global carbon dioxide flux in soil respiration and its relationship to vegetation and climate. Tellus B 44: 81–99.

[10] Archer D (2010) The Global Carbon Cycle. Princeton University Press (Princeton, NJ, USA).

[11] Whalen SC (2005) Biogeochemistry of methane exchange between natural wetlands and the atmosphere. Environ. Eng. Sci. 22: 73–94.

[12] Mitsch WJ, Bernal B, Nahlik AM, Mander Ü, Zhang L, Anderson CJ, et al (2013) Wetlands, carbon, and climate change. Landscape Ecol. 28: 583–597.

[13] Petrescu AMR, Lohila A, Tuovinen J-P, Baldocchi DD, Desai AR, Roulet NT, et al (2015) The uncertain climate footprint of wetlands under human pressure. Proc. Nat. Acad. Sci. 112: 4594–4599. 10.1073/pnas.1416267112 25831506 PMC4403212

[14] Myhre G, Shindell D, Bréon F-M, Collins W, Fuglestvedt J, Huang J, et al (2013) Anthropogenic and natural radiative forcing, in *Climate Change 2013*: *The Physical Science Basis*, edited by Stocker T.F., Qin D., Plattner G. -K., Tignor M., Allen S. K., Boschung J., Nauels A., Xia Y., Bex V., and Midgley P. M., pp. 659–740, Cambridge, UK and New York, NY, USA: Cambridge University Press.

[15] Whiting GJ, Chanton JP (1993) Primary production control of methane emissions from wetlands. Nature 364: 794–795.

[16] Poffenbarger HJ, Needleman BA, Megonigal JP (2011) Salinity influence on methane emissions from tidal marshes. Wetlands 31: 831–842.

[17] Pangala SR, Moore S, Hornibrook ERC, Gauci V (2013) Trees are major conduits for methane egress from tropical forested wetlands. New Phytol. 197: 524–531. 10.1111/nph.12031 23253335

[18] Holm GO Jr, Perez BC, McWhorter DE, Krauss KW, Johnson DJ, Raynie RC, et al (2016) Ecosystem level methane fluxes from tidal freshwater and brackish marshes of the Mississippi River Delta: Implications for coastal wetland carbon projects. Wetlands 36: 401–413.

[19] Neubauer SC, Megonigal JP (2015) Moving beyond global warming potentials to quantify the climatic role of ecosystems. Ecosystems 18: 1000–1013.

[20] Searchinger T, Heimlich R., Houghton RA, Dong F, Elobeid A, Fabiosa J, et al (2008) Use of U.S. croplands for biofuels increases greenhouse gases through emissions from land-use change. Science 319: 1238–1240. 10.1126/science.1151861 18258860

[21] Kim H, Kim S, Dale BE (2009) Biofuels, land use change, and greenhouse gas emissions: Some unexplored variables. Environ. Sci. Technol. 43: 961–967. 19245043 10.1021/es802681k

[22] Gleason RA, Euliss NH Jr, Tangen BA, Laubhan MK, Browne BA (2011) USDA conservation program and practice effects on wetland ecosystem services in the Prairie Pothole Region. Ecol. Appl. 21: S65–S81.

[23] Hertel TW, Golub AA, Jones AD, O’Hare M, Plevin RJ, Kammen DM (2010) Effects of US maize ethanol on global land use and greenhouse gas emissions: Estimating market-mediated responses. BioScience 60: 223–231.

[24] Knox SH, Sturtevant C, Hatala Matthes JA, Koteen L, Verfaillie J, Baldocchi D (2015) Agricultural peatland restoration: Effects of land-use change on greenhouse gas (CO_2_ and CH_4_) fluxes in the Sacramento-San Joaquin Delta. Glob. Change Biol. 21: 750–765. 10.1111/gcb.12745 25229180

[25] Hatala JA, Detto M, Sonnentag O, Deverel SJ, Verfaillie J, Baldocchi DD (2012) Greenhouse gas (CO_2_, CH_4_, H_2_O) fluxes from drained and flooded agricultural peatlands in the Sacramento-San Joaquin Delta. Agr. Ecosyst. Environ. 150: 1–18.

[26] Hendriks DMD, van Huissteden J, Dolman AJ, van der Molen MK (2007) The full greenhouse gas balance of an abandoned peat meadow. Biogeosciences 4: 411–424.

[27] Meijide A, Manca G, Goded I, Magliulo V, di Tommasi P, Seufert G, et al (2011) Seasonal trends and environmental controls of methane emissions in a rice paddy field in Northern Italy. Biogeosciences 8: 3809–3821.

[28] Tian H, Lu C, Ciais P, Michalak AM, Canadell JG, Saikawa E, et al (2016) The terrestrial biosphere as a net source of greenhouse gases to the atmosphere. Nature 531: 225–228. 10.1038/nature16946 26961656

[29] Schlesinger WH, Andrews JA (2000) Soil respiration and the global carbon cycle. Biogeochem. 48: 7–20.

[30] Brix H, Ye S, Laws EA, Sun D, Li G, Ding X, et al (2014) Large-scale management of common reed, *Phragmites australis*, for paper production: A case study from the Liaohe Delta, China. Ecol. Eng. 73: 760–769.

[31] Mou X, Sun Z, Wang L, Wang C (2011) Nitrogen cycle of a typical *Suaeda salsa* marsh ecosystem in the Yellow River estuary. J. Environ. Sci. 23: 958–967. 10.1016/s1001-0742(10)60530-x 22066219

[32] Brix H, Sorrell BK, Lorenzen B (2001) Are *Phragmites*-dominated wetlands a net source or net sink of greenhouse gases? Aqua. Bot. 69: 313–324.

[33] Xu X, Zou X, Cao L, Zhamangulova N, Zhao Y, Tang D, et al (2014) Seasonal and spatial dynamics of greenhouse gas emissions under various vegetation covers in a coastal saline wetland in southeast China. Ecol. Eng. 73: 469–477.

[34] Sun Z, Wang L, Tian H, Jiang H, Mou X, Sun W (2013) Fluxes of nitrous oxide and methane in different coastal *Suaeda salsa* marshes of the Yellow River estuary, China. Chemosphere 90: 856–865. 10.1016/j.chemosphere.2012.10.004 23134757

[35] Chen Q, Ma J, Liu J, Zhao C, Liu W (2013) Characteristics of greenhouse gas emissions in the Yellow River Delta wetland. Int. Biodeter. Biodegr. 85: 646–651.

[36] Chen Q, Ma J, Zhao C, Li R (2015) The spatial and temporal variation characteristics of CH_4_ and CO_2_ emission flux under different land use types in the Yellow River Delta wetland. J. Geosci. Environ. Prot. 3: 26–32.

[37] Song H, Liu X (2016) Anthropogenic effects on fluxes of ecosystem respiration and methane in the Yellow River Estuary, China. Wetlands 36: 113–123.

[38] Olsson L, Ye S, Yu X, Wei M, Krauss KW, Brix H (2015) Factors influencing CO_2_ and CH_4_ emissions from coastal wetlands in the Liaohe Delta, Northeast China. Biogeosci. 12: 4965–4977.

[39] Curial Yuste J, Janssens IA, Carrara A, Ceulemans R (2004) Annual Q_10_ of soil respiration reflects plant phenological patterns as well as temperature sensitivity. Glob. Change Biol. 10: 161–169.

[40] Dinsmore KJ, Skiba UM, Billett MF, Rees RM (2009) Effect of water table on greenhouse emissions from peatland mesocosms. Plant Soil 318: 229–242.

[41] Krauss KW, Whitbeck JL, Howard RJ (2012) On the relative roles of hydrology, salinity, temperature, and root productivity in controlling soil respiration from coastal swamps (freshwater). Plant Soil 358: 265–274.

[42] Matson PA, Harriss RC (1995) Biogenic Trace Gases: Measuring Emissions from Soil and Water. Blackwell Science Ltd.

[43] Mao R, Zhang X, Meng H (2014) Effect of *Suaeda salsa* on soil aggregate-associated organic carbon and nitrogen in tidal salt marshes in the Liaohe Delta, China. Wetlands 34: 189–195.

[44] Bartlett KB, Bartlett DS, Harriss RC, Sebacher DI (1987) Methane emissions along a salt marsh salinity gradient. Biogeochem. 4: 183–202.

[45] Huang G, Li X, Hu Y, Shi Y, Xiao D (2005) Methane (CH_4_) emissions from a natural wetland of northern China. J. Environ. Sci. Health 40: 1227–1238. 10.1081/ese-200055666 15921278

[46] Megonigal JP, Schlesinger WH (2002) Methane-limited methanotrophy in tidal freshwater swamps. Global Biogeochem. Cycles 16: 1088.

[47] Yan X, Cai Z, Ohara T, Akimoto H (2003) Methane emission from rice fields in mainland China: Amount and seasonal and spatial distribution. J. Geophys. Res. 108: 4505.

[48] Zou J, Huang Y, Jiang J, Zheng X, Sass RL (2005) A 3-year field measurement of methane and nitrous oxide emissions from rice paddies in China: Effects of water regime, crop residue, and fertilizer application. Glob. Biogeochem. Cycles 19: GB2021.

[49] World Meteorological Organization (2014) WMO Statement on the Status of the Global Climate in 2013. World Meteorological Organization, WMO-No.1130, Geneva, Switzerland. 21 p.

[50] Song W, Wang H, Wang G, Chen L, Jin Z, Zhuang Q, et al (2015) Methane emissions from an alpine wetland on the Tibetan Plateau: Neglected but vital contribution of the nongrowing season. J. Geophys. Res. Biogeosci. 120: 1475–1490.

[51] Ehhalt D, Prather M, Dentener F, Derwent R, Dlugokencky E, Holland E, et al (2001) Atmospheric chemistry and greenhouse gases, in *Climate Change 2001*: *The Scientific Basis*, edited by Houghton J.T., Ding Y., Griggs D.J., Noguer M., van der Lindon P.J., Dai X., Maskell K., and Johnson C.A., pp. 239–287, Cambridge, UK: Cambridge University Press.

[52] Global Carbon Project (2013) Methane Budget and Trends 2013. http://www.globalcarbonproject.org/methanebudget. Accessed 3 December 2015.

[53] Yu KW, Faulkner SP, Patrick WH (2006) Redox potential characterization and soil greenhouse gas concentration across a hydrological gradient in a Gulf Coast forest. Chemosphere 62: 905–914. 16043211 10.1016/j.chemosphere.2005.05.033

[54] Zhu X, Song C, Guo Y, Sun X, Zhang X, Miao Y (2014) Methane emissions from temperate herbaceous peatland in the Sanjiang Plain of Northeast China. Atmos. Environ. 92: 478–483.

[55] Zhang H, Zhao X, Ni Y, Lu X, Chen J, Su F, et al (2010) PCDD/Fs and PCBs in sediments of the Liaohe River, China: Levels, distribution, and possible sources. Chemosphere 79: 754–762. 10.1016/j.chemosphere.2010.02.039 20236682

[56] Zhu L, Wu J, Xu Y, Hu R, Wang N (2010) Recent geomorphic changes in the Liaohe Estuary. J. Geogr. Sci. 20, 31–48.

[57] Livingston GP, Hutchinson GL (1995) Enclosure-based measurement of trace gas exchange: applications and sources of error, In Matson P. A. and Harriss R. C. [Eds.], Biogenic Trace Gases: Measuring Emissions from Soil and Water. Blackwell Science Ltd pp. 14–46.

[58] Neubauer SC, Miller WD, Anderson IC (2000) Carbon cycling in a tidal freshwater marsh ecosystem: a carbon gas flux study. Mar. Ecol. Prog. Ser. 199: 13–30.

[59] Tong C, Huang JF, Hu ZQ, Jin YF (2013) Diurnal variations of carbon dioxide, methane, and nitrous oxide vertical fluxes in a subtropical estuarine marsh on neap and spring tide days. Estuaries Coasts 36: 633–642.

[60] Craft CB (2007) Freshwater input structures soil properties, vertical accretion, and nutrient accumulation of Georgia and U.S. tidal marshes. Limnol. Oceanogr. 52: 1220–1230.

[61] McKee KL, Mendelssohn IA, Hester MW (1988) Reexamination of pore water sulfide concentrations and redox potentials near the aerial roots of *Rhizophora mangle* and *Avicennia germinans*. Am. J. Bot. 75: 1352–1359.

[62] Johnson RA, Wichern DW (1988) Applied Multivariate Statistical Analysis, 2nd Ed., Prentice Hall (New Jersey, USA).

[63] Krauss KW, Whitbeck JL (2012) Soil greenhouse gas fluxes during wetland forest retreat along the lower Savannah River, Georgia (USA). Wetlands 32: 73–81.

[64] Zona D, Gioli B, Commane R, Lindaas J, Wofsy SC, Miller CE, et al (2016) Cold season emissions dominate the Arctic tundra methane budget. Proc. Nat. Acad. Sci. 113: 40–45. 10.1073/pnas.1516017113 26699476 PMC4711884

