# Supplementary material for: Inter-Annual Variability of Area-Scaled Gaseous Carbon Emissions from Wetland Soils in the Liaohe Delta, China
Source: PLoS One. 2016 Aug 8;11(8):e0160612. doi: 10.1371/journal.pone.0160612 (PMC4976977; doi:10.1371/journal.pone.0160612)
Supplement: S1 Table — Data are updated from [34] to include additional data collected in 2013 and 2014. (DOCX) [file pone.0160612.s001.docx]

**S1 Table.** Soil characteristics from a depth of 0-10 cm at five wetland sites in the Liaohe Delta, China. Data are updated from [38] to include additional data collected in 2013 and 2014.

|  |  |  |  |  |  |
| --- | --- | --- | --- | --- | --- |
| **Soil characteristic** | **Suaeda1** | **Suaeda2** | **Phrag1** | **Phrag2** | **Rice** |
| Bulk density (g/cm^3^) | 1.41 ± 0.03 | 1.33 ± 0.05 | 1.30 ± 0.07 | 0.65 ± 0.12 | 1.45 ± 0.03 |
| Moisture content (%) | 24.1 ± 0.3 | 25.8 ± 0.6 | 26.7 ± 1.5 | 49.2 ± 6.5 | 24.6 ± 0.6 |
| N density (mg N/cm^3^) | 1.03 ± 0.02 | 1.04 ± 0.04 | 1.92 ± 0.10 | 3.85 ± 0.71 | 1.20 ± 0.02 |
| C density (mg C/cm^3^) | 12.55 ± 0.27 | 11.32 ± 0.43 | 19.50 ± 1.05 | 46.33 ± 8.55 | 10.22 ± 0.21 |
| C:N mass ratio | 12.2 | 10.9 | 10.2 | 12.0 | 8.5 |
| Organic C density (mg C/cm^3^) | 6.56 ± 0.14 | 8.1 ± 0.30 | 16.12 ± 0.87 | 42.44 ± 7.84 | 8.56 ± 0.18 |
| Organic C / N (mol:mol) | 7.4 ± 0.3 | 9.3 ± 1.5 | 9.6 ± 1.2 | 11.8 ± 1.0 | 8.5 ± 0.3 |
